# Home-schooling and caring for children during the COVID-19 lockdown in the UK: emotional states, systems of support and coping strategies in working mothers

**DOI:** 10.3389/fsoc.2024.1168465

**Published:** 2024-03-21

**Authors:** Angeliki Kallitsoglou, Pamela-Zoe Topalli

**Affiliations:** ^1^School of Education, University of Exeter, Exeter, United Kingdom; ^2^Department of Teacher Education, University of Turku, Turku, Finland

**Keywords:** COVID-19, lockdown, working mothers, home-schooling, childcare, maternal wellbeing, emotional states, United Kingdom

## Abstract

**Introduction:**

We examined the experience of the intensification of home-schooling and/or childcare in working mothers in the United Kingdom during the first national COVID-19 lockdown. Our focus was on understanding how mothers dealt with this challenging period both emotionally and practically.

**Methods:**

Eligible mothers (*n* = 47; Mage  =  39.6) participated in an anonymous online survey of openended questions.

**Results:**

Thematic analysis of responses showed that mothers found home-schooling and/or childcare to be challenging. This was particularly notable in situations where support from partners, schools, and workplaces was limited. For single working mothers, the absence of support resources was especially impactful. Mothers often felt overly stressed trying to balance work and family responsibilities, guilty for not meeting their child’s needs, and were worried over their child’s well-being and academic progress and over increasing work demands. Common strategies mothers used to cope with the challenges of home-schooling and/or childcare included adopting a positive outlook, implementing flexible family structures, increasing family connectedness, and negotiating alternative partnership models.

**Discussion:**

The intensification of home-schooling and/or childcare during the lockdown in the United Kingdom negatively affected maternal well-being, particularly due to limited support. These findings underscore the importance of prioritizing maternal wellbeing in post-pandemic recovery efforts. Additionally, they highlight the social dimension of maternal wellbeing and suggest a comprehensive approach to support it that includes both timely access to intervention for mental health but also implementing family-friendly work policies and offering support with childcare and children’s learning as essential measures.

## 1 Introduction

In March 2020, many countries implemented country-wide quarantine measures, commonly referred to as lockdowns, to mitigate the impact of the novel coronavirus (COVID-19) on public health. The COVID-19 lockdowns led to prolonged school and daycare center closures. Consequently, most caregivers had no option but to home-school their children and/or spend more time looking after them. It is estimated that the increase in the volume of hours spent on unpaid childcare during lockdown ranged from 25% in Spain, to 37% in Hungary, and up to 50% in the United Kingdom (Derndorfer et al., 2021). Mothers were disproportionately affected by school closures, and loss of childcare and social support. It has been extensively reported that mothers shouldered the bulk of unpaid domestic work, even when their partner worked from home (e.g., Daniela et al., 2021; Harrop, 2021; Meraviglia and Dudka, 2021). The home-schooling and steep increase in caring responsibilities during the lockdowns put mothers at risk of poor mental health and wellbeing (e.g., Taylor et al., 2021; Racine et al., 2022). The impact of the loss of formal and informal support was more dramatic for lone-working mother households because of the additional strain on overstretched physical, emotional, and/or financial reserves (Arnot, 2020).

The significant impact of the pandemic on the United Kingdom population led to school and nursery closures on 20 March 2020, except for the children of key workers and vulnerable children for whom settings remained open as a ‘childminding’ service (Khan, 2022). A general lockdown was implemented on 23 March 2020. As part of the relaxation of the lockdown measures from June 1 2020 primary schools started a phased re-opening limited to three groups: Reception, Year 1, and Year 6. Nurseries and other childcare facilities for preschool children were allowed to open from this time (Chung et al., 2021). In the break of the lockdown in the United Kingdom in 2020, census data showed that mothers were more likely than fathers to undertake childcare responsibilities (Office for National Statistics, 2020b) and report poor mental wellbeing (Office for National Statistics, 2020a). The intensification of domestic responsibilities in mothers raised concerns about the wellbeing of working mothers. As a result, there has been a growing interest in the lockdown experiences of working mothers in the United Kingdom (Adisa et al., 2021; Hoskins and Wainwright, 2023). However, there is less attention to working mothers’ experiences that are specific to homeschooling and/or caring for children while working (H/CWW). The aim of the present study was to examine working mother’s inner experiences of H/CWW during the COVID-19 school closures in the United Kingdom, their emotional responses to these experiences, and the ways they coped practically and emotionally. This examination will help better understand the role of H/CWW in maternal mental wellbeing during the pandemic lockdown in the United Kingdom. Additionally, it will help inform targeted health and social policy interventions for post-pandemic recovery and for working mothers’ mental wellbeing in the face of future public health crisis and adversity.

## 2 The intensification of childcare responsibilities and home-schooling in mothers during the COVID-19 lockdown in the United Kingdom

Home-schooling and caring for children during the pandemic had had an unprecedented negative impact on caregiver mental wellbeing. Several studies have shown that more time spent in home-schooling was linked to higher levels of stress (Thorell et al., 2021; Cuadrado et al., 2022; May and Hoerl, 2022), worry (Thorell et al., 2021), anxiety and depression (Deacon et al., 2021), loneliness (Cuadrado et al., 2022), and decreased positive affect (Heers and Lipps, 2022). Moreover, more time spent on childcare was associated with increased levels of psychological distress (Xue and McMunn, 2021). Studies that allowed for comparison between men and women showed that women often were more overwhelmed with home-schooling (Heers and Lipps, 2022). Additionally, they experienced a heavier mental load about domestic responsibilities (Czymara et al., 2020) and spent more time home-schooling and caring for children (Andrew et al., 2020b; Derndorfer et al., 2021; Xue and McMunn, 2021). Even when paternal involvement in unpaid domestic work increased, mothers still undertook a higher share of domestic responsibilities (Derndorfer et al., 2021). The experience of home-schooling/caring was particularly challenging for working mothers because they had to juggle competing roles simultaneously and with limited support (Di Giorgio et al., 2020; Kapoor et al., 2021; Xue and McMunn, 2021).

International and European indicators of gender equality suggest that the United Kingdom is a country friendly toward gender egalitarianism. The Global Index of Gender Equality of the World Economic Forum (WEF) shows benchmark progress toward gender parity by comparing countries’ gender gaps across four dimensions: economic opportunities, education, health and political leadership (World Economic Forum, 2023). Eligible countries for inclusion into the index include leading as well as developing economies. In line with recent data, the United Kingdom is top 15 out of 146 countries in gender parity (score = 0.79; range: 0 = no parity – parity = 1) (World Economic Forum, 2023). The European equivalent is the Gender Equality Index (GEI) which measures gender equality in five core domains: work, money, knowledge, time, power and health (European Institute for Gender Equality, 2020). The United Kingdom has been topping the EU GEI since 2010, and in 2020 was ranked 6th out of 28 EU countries (European Institute for Gender Equality, 2020). Regarding public opinion, a large global survey on gender equality showed that in EU and North American region the United Kingdom was amongst the countries with the highest rate of public endorsement of the move toward greater gender equality (Poushter et al., 2019).

Despite progress toward gender egalitarianism in British society, mothers shouldered more of the extra childcare responsibilities during the United Kingdom lockdown. Real-time data on daily lives from a large sample of United Kingdom families with children under the age of 12 showed that families were doing the equivalent of a working week in childcare (i.e., physical care and supervision, feeding, teaching, reading, talking, and accompanying the child to do activities among others) (Sevilla and Smith, 2020). The mothers bore most of the childcare irrespective of their employment status. Data from the United Kingdom Office for National Statistics (ONS) showed that during the 2020 lockdown mothers overall spent more time on both developmental and non-developmental childcare, such as reading a book and dressing, respectively (Office for National Statistics, 2020b). The time mothers spent on non-developmental childcare exceeded that of men by 77%. Additionally, of all the caregivers who were home-schooling, one in three women felt that it was negatively affecting their well-being compared with one in five men (Office for National Statistics, 2020a). These trends raise concerns about the impact of the intensification of childcare and home-schooling on the mental wellbeing of working mothers during the lockdown and in the long-term.

### 2.1 The emotional experience of home-schooling and childcare while working during the COVID-19 lockdown

The specification of the emotions triggered in response to the events of H/CWW can enhance our understanding of the social phenomenon of H/CWW and its consequences on maternal mental wellbeing. Emotions often represent emotional states, such as fear, anger, or joy in response to either external reinforcing events, the recall or appraisal of reinforcing events (Rolls, 2013). Negative emotional states such as feeling nervous, sad, or anxious are indicators of psychopathology (Spielberger, 2006). Positive emotions, on the other hand, are related to increased physical and emotional wellbeing such as resilience to stress, improved physical health and quality of life, and a higher level of psychological reserves (Fredrickson, 2001; Cohn et al., 2009; Gloria and Steinhardt, 2016).

Emotions are not isolated from the social reality in which they are experienced. From a sociological perspective, human emotions emerge, are experienced, and have meaning in the context of specific social situations (Bericat, 2016). For instance, previous research has linked specific emotional states with social phenomena such as social order (Srbljinović and Božić, 2014) and intensive mothering (Rodriguez Castro et al., 2022). Therefore, understanding the social context of emotions can increase our understanding of both human emotions and the complexity of the social processes that generate them. Equally, social phenomena are not independent of human emotions. The expressive and motivational nature of emotions makes them an important component of sociability (Bericat, 2016). Hence, the integration of the affective dimension into the investigation of social phenomena can help better understand the social world (Verweij et al., 2015).

Emotions are indirect manifestations of our inner emotional experiences. Capturing the individual’s subjective emotional experience of an event can help manifest the specific emotional state related to this event (Robinson and Clore, 2002). The research links several H/CWW related experiences that might have influenced working mothers’ mental wellbeing. These include role strain and role conflict (Lutz et al., 2022), intensive mothering (Aslam and Adams, 2022), a ‘work-first’ organizational culture, the gendered distribution of household responsibilities (Zanhour and Sumpter, 2022) and the mother as a teacher (Hoskins and Wainwright, 2023). The examination of the affective dimension of the H/CWW experience can help understand the specific emotional states that were triggered by these experiences and vice versa.

### 2.2 Working mothers’ emotional experience of home-schooling and childcare during the United Kingdom COVID-19 lockdown

There is great variability in maternal lockdown experiences and associated emotional responses (Brom et al., 2020; Brooks et al., 2020; Settersten et al., 2020; Crisci et al., 2021; Van Bakel et al., 2022). Much of the evidence base on working mothers’ experience during the lockdown in the United Kingdom has not considered systematically the experience of H/CWW and the associated emotions. For instance, a qualitative study of British working mothers showed that keeping a family-work life balance was challenging but the mothers appreciated the opportunity for increased family closeness and rediscovery of family values (Adisa et al., 2021). However, the study did not specifically examine the home-schooling and childcare experience and how mothers felt about it. A qualitative study of a sample of six low-income mostly working single mothers living in London, United Kingdom reported that undertaking the teacher’s role was a difficult experience both practically and emotionally (Hoskins and Wainwright, 2023). Participants reported that they had to teach material that they did not understand themselves which caused worry and anxiety. However, the findings cannot be extrapolated to partnered working mothers. Additionally, the study did not report maternal education which could be implicated in the mothers’ anxiety to enact the teacher role.

Another group of studies on home-schooling/childcare during the lockdown does not focus on working mothers specifically. This methodological intricacy prevents us from understanding the unique home-schooling/childcare experiences of working mothers. For instance, a qualitative study of caregivers living in London, United Kingdom found that home-schooling/childcare was associated with both positive and negative experiences (Khan, 2022). These included excitement by the prospect of having children at home, feeling overwhelmed and stressed because of home-schooling, insecurity with supporting children’s learning, difficulty dealing with the dual role of parent-teacher, and a loss of identity. However, the study samples included both mothers and fathers and a mixture of working (remote and non-remote) and not working parents. A large sample of highly ethnically diverse mothers living in England answered open-ended questions about their mental health during the lockdown (Dickerson et al., 2020). The study showed that the mothers experienced health-related anxieties, high mental load of managing multiple roles and responsibilities, loss of social support and other coping strategies, financial pressures, employment insecurity, and difficulties to switch off from the pandemic. However, most of the mothers were not working and no questions specifically about the experience of H/CWW were examined.

Another limitation of the existing literature is the little attention to the coping strategies specific to home-schooling and caring for children during the lockdown in working mothers in the United Kingdom. Positive adaptation to stressful situations has protective qualities for mental wellbeing (Jones et al., 2022; Alat et al., 2023). Therefore, as well as the emotional experience of H/CWW, understanding the ways working mothers coped with those experiences can help understand better the state of their mental wellbeing during the lockdown. Existing studies on the home-schooling and/or caregiving experience among parents in the United Kingdom showed that better coping strategies were related to better socioemotional adjustment. For instance, Khan (2022) found that taking a pragmatic approach and tapping on parent support networks helped a few working mothers combat the stress. A study of a sample with a high representation of home-schooling mothers living in the United Kingdom during the lockdown showed that caregiver self-efficacy and low self-blame were associated with positive reappraisal (Aznar et al., 2021). In a sample of predominantly maternal and home-working caregivers living in Portugal and United Kingdom showed that a greater appreciation of life and discovering and embracing new possibilities were associated with more optimal well-being (Stallard et al., 2021). A qualitative study of British working mothers on work-family life balance during the lockdown showed that mothers appreciated the opportunity for increased family closeness and rediscovery of family values (Adisa et al., 2021).

Finally, several of the earlier studies that examined the home-schooling/childcare experience in caregivers in the United Kingdom (for an example see Thorell et al., 2021 and Aznar et al., 2021) and maternal mental wellbeing (for an example see Stallard et al., 2021) often use maternal scores on a self-reported rating scale. However, this approach could miss capturing the specific experience that triggered specific emotional responses to H/CWW. In this instance, a qualitative approach is appropriate because it can provide mothers with the space to give their own reasons of how and why they experienced the home-schooling/childcare situation in the way they did. Additionally, it can enhance our understanding about what it was like home-schooling/caring for children for working women by accessing the fine details of their lived experience.

### 2.3 The present study

The aim of the present study was to capture the experiences of home-schooling and/or childcare during the United Kingdom COVID-19 lockdown in working mothers, the emotions associated with these experiences and the ways working mothers coped practically and emotionally. The study adds to earlier research that may have missed capturing the emotions specifically related to the experience of home-schooling/caring for children in working mothers and in the context of the lockdown in the United Kingdom in 2020. Based on existing research, we expected that mothers were likely to have experienced various practical and emotional challenges reported in the previous literature such as increased mental load, role strain, physical and mental exhaustion, loss of support, unequal distribution of domestic labor and taking up the teacher’s role. Additionally, we expected that mothers would share a range of adaptive coping strategies such as positive reappraisal, acceptance, adopting a pragmatic approach, greater appreciation of life, and embracing new possibilities.

## 3 Methods

### 3.1 Participants

Fifty-five working mothers consented to complete an anonymous electronic survey distributed between June and the first week of August 2020 via social media. These included mother groups on Facebook and social networking platforms such as LinkedIn and Whatsapp. The data of those who were not residing in the United Kingdom during the lockdown (*n* = 1), were not working (*n* = 5), did not provide evidence of whether they were working (*n* = 1) and those with incomplete questions (*n* = 1) were not included in the study. The mothers in the final sample (*n* = 47) were primarily white (*n* = 38, 81%) working mothers (*n* = 25, 53% full-time; *n* = 40, 85% working from home) between 28 and 54 years old (*M*_age_ = 39.6 *SD* = 5.65). Children were between 1 to 17 years old. Nearly two in three households (70%) were homeschooling children and 57% of the households had more than one child. Most mothers had at least a higher education degree (*n* = 40, 85%) and were married (*n* = 35, 75%). The study did not ask mothers to report their sex or that of their partner but maternal responses to the questions indicated that they were female and married or had been married into heterosexual partners. Ethical approval was granted by the Ethics Committee at the University of Turku, Finland, Humanities and Social Sciences Division.

### 3.2 Measures and procedures

#### 3.2.1 Survey development

The second author and her colleagues (Topalli et al., 2020) developed an anonymous survey of 20 questions to examine working and studying mothers’ experiences of the COVID-19 lockdown and associated feelings. The survey was developed in three languages: English, Finish and Greek. The developers had experience of home-schooling/looking after children while working and/or studying from home during the first lockdown in Finland in 2020. The survey was reviewed by the developers’ international network of peers with experience in social science research and their comments were incorporated in the final survey. The English version was reviewed by the first author who was working remotely and had home-schooling and childcare responsibilities during the first national lockdown in the United Kingdom in the Spring of 2020. The English version was adapted to include a question on ethnicity. The online platform Webropol^1^ was used to develop and disseminate an electronic version of the survey. A copy of the survey is provided in Supplementary file 1.

The survey started by introducing the study and inviting the participants to take part. After having read the information, participants were asked to consent to participate before proceeding. Once consent was provided, the participants were invited to share details of basic sociodemographic information (i.e., maternal age, work mode, education level completed, number of children in the household, child/children’s age, family structure, mother’s workplace during the state of emergency, partner’s workplace during the state of emergency, country of residence during the state of emergency, child’s schooling situation during the state of emergency, and COVID-19 high risk group membership).

The survey continued with 6 open-ended questions designed to capture how working mothers experienced the challenges of having to care for children and homeschool while working and how they coped emotionally and practically. A summary of the questions is provided below: (1) general mood, thoughts and feelings (e.g., *how has your mood been in general?*); (2) working life (e.g., *how has working affected your mood and feelings?*); (3) social life (e.g., *how has your social life changed? how have those changes affected your mood and feelings?*); (4) daily life (e.g., *how has your everyday life changed? how have those changes affected your mood and feelings?*); (5) combining work with childcare, home-schooling, and homecare (e.g., *how have you experienced the combination of work, managing kids’ remote schooling, and childcare? what factors have made it easier and/or harder to handle? What thoughts and feelings has this situation brought to you?*); (6) thoughts and feeling about the society opening up/returning to normal (e.g., *now that the society is starting to slowly open again, what kinds of thought and feelings has it brough to you? How has it affected your mood?*).

#### 3.2.2 Analysis

We used thematic analysis which allows the identification of themes within the data (Braun and Clarke, 2006). A theme refers to pieces of information in the data that reflect patterned responses or meanings, and are relevant to the research question (Braun and Clarke, 2006). Patterned responses which are linked under a common idea are assigned codes which are then grouped under major themes. We used a 6 -step guide of conducting thematic analysis described in Braun and Clarke (2006): familiarizing with the data; initial code generation; theme development; theme review; theme definition; report production. Accordingly, first, the authors read the participants’ answers to the 6 open-ended questions to identify meaningful units of text that were relevant to the research aims (i.e., experience of H/CWW and associated emotions and coping). Second, they grouped together units of text dealing with the same topic to form categories which they then gave provisional definitions. The same unit of text could be included in more than one category. Then, the authors reviewed the categories to ensure that the data identified for each one was exhaustive and that it reflected the definition of the category that it was grouped under.

The first author used the NVivo 12 software^2^ for qualitative data analysis to carry out the primary coding of maternal responses to the 6 open-ended questions. The analysis yielded several codes that were grouped into themes. To examine the reliability of the initial themes the second author coded all the data. The authors did not rely on *a priori* analytical framework, nor did they use the questions as a framework, but attempted to allow the data to drive the coding. Therefore, before coding the data, a discussion between authors took place on how to approach the coding.

To enhance understanding of the maternal emotional states related specifically with the experience of H/CWW the thematic analysis was supplemented with a word frequency analysis. This examination involves screening maternal extracts selected to inform the thematic analysis for words that reflect emotional states (e.g., *felt sad*; *feelings of frustration; I was angry; felt pride*). The number of times each word and its derivative (sad/sadness) appear is counted to create a total word count. The findings can be used to visualize the range and frequency of the different emotional states experienced by the participants.

## 4 Findings

The initial analysis carried out by the first author resulted in 19 categories which were grouped into six key themes: *the experience of home-schooling and/or childcare, maternal emotional states, coping strategies, support, delivering and managing home-schooling, and child wellbeing and academic achievement.* The second author identified four themes that closely matched those identified by the first author: *maternal emotional state*, *coping strategies*, *managing challenges with home-schooling and/or childcare*, and *challenges with home-schooling*. A discussion between the two coders led to the following theme reduction decisions. The categories of the *support* theme identified by the first author were instead diffused across the different themes identified by the second author. Because of its prominence it was decided to keep *support* as a theme of its own. The themes relevant to the challenges with home-schooling identified by both authors were incorporated into the *experience of home-schooling and/or childcare* theme. Finally, the theme *child wellbeing and academic achievement* identified by the first author was initially included as a category of the theme *managing challenges with home-schooling and/or childcare* identified by the second author. Because the common thread in this theme was *worry* about children’s wellbeing and achievement it was incorporated in the *emotional states* theme. The final four themes were*: the experience of home-schooling and/or childcare, emotional states, support,* and *adaptive coping strategies.*

### 4.1 The experience of home-schooling and/or childcare while working

The analysis showed that mothers found the experience of H/CWW challenging. The difficulties left many physically and mentally exhausted and with little or no time to *switch off* as a full-time home-working mother with a young child (M19) described:

*I’ve worked all the hours in between looking after the little one and the house work and the life admin and the food shopping. I’ve worked till 10 pm at night to write reports, checked emails at 7 am or on the go (they go straight to my personal mobile so I cant switch off). I feel conflicted all the time, like I cant do it all but people around me seem to do it all and if I drop the ball on something I feel like I’ve failed. I want to be a great mum and great at my job and the cost comes to my personal wellbeing.*

Historically, mothers have been undertaking the bulk of domestic responsibilities, but supporting the home-schooling process while working added a new dimension to an already heavy daily routine.

*I’ve always had to juggle working full time and looking after the children [….] what compounded my feelings of frustration has been the home schooling*. (M30; Full-time homeworking partnered mother of two children).

*I am suffering from ‘home school’ fatigue*. (M32; Part-time homeworking single mother of one child).

What seemed to be most challenging about home-schooling for both partnered and single mothers was having to deliver it while working. Synchronous delivery often involved managing conflicting demands and handling constant interruptions and unforeseeable events such as child emotional outbursts and sibling fights.

*I have been working normal hours and had my usual caseload. On top of that I had to look after my son, so often I would be in a session and at the same time had to make sure my son does not climb on the sofa and falls down!* (M03; Full-time homeworking partnered mother of a young child).

Difficulty with synchronous home-schooling delivery was intensified by child characteristics including age, special educational needs (SEN), temperament, and motivation. For instance, young children needed more support to engage with homework or activities. SEN provision (i.e., dyslexia, dyspraxia, attention deficit and hyperactivity, and autism) was not always easy to be implemented remotely. Children described by mothers as demanding, constantly seeking attention, and showing reluctance toward schoolwork made the delivery and management of home-schooling particularly difficult. Many mothers reported that keeping their children motivated and engaged with home learning was not an easy task. The lack of motivation and engagement hindered the delivery and management of home-schooling. Another group of mothers shared that despite starting energized and motivated to home-school gradually they lost motivation or got tired with it. Lack of access to electronic devices and the internet was another reason shared as intensifying the difficulty in delivering home-schooling.

Undertaking the teachers’ role posed additional challenges. A few mothers reported low confidence and competence in teaching their children. This finding was unrelated to having spent fewer years in education as they all had at least a higher education degree. They also felt that undertaking the role of the teacher was frustrating both for the child and the mother because it competes the mothering role. Moreover, lack of time for preparation to explain the material and high volume of homework made the experience of home-schooling more difficult.

*Its been so so so hard. I am not a teacher, my daughter wants me to be her Mum and not her teacher - this has been learnt the hard way through upset and tears and tantrums (hers and mine!)*. (M19; Full- time homeworking partnered mother with one child).

*I find it frustrated, teachers put too much homework, without thinking about parents who are working, is not my job to teach and I had to go and read things before I explained them to my son*. (M10; Full-time working out of home partnered mother of two children).

When we looked at the experiences of single mothers only (*n* = 12) we found that they were all challenged juggling competing demands of H/CWW alone. The home-schooling/childcare demands interacted with personal characteristics such as type of employment (e.g., freelancing), key worker status, or additional caregiving responsibilities. The interaction occasionally added an extra layer of challenge. For instance, a mother working as a freelancer and having to look after elderly parents expressed how she ended up losing clients.

*I was helping both with school work till just before summer half term but my work suffered and I lost a client. It has been a terrible situation made worse by stresses of living with elderly parents*. (M31: Part-time homeworking single-mother of two children).

On a positive note, a few mothers saw the benefits in supporting children’s learning, despite the difficulties with H/CWW.

*What I am happy for is the time I have regained with my son because we are around each other all the time. I feel I understand where he is academically a lot better so know where to help more*. (M18; Full-time homeworking single-mother of one child).

### 4.2 Emotional states

Mothers experienced a wide range of negative emotions with the most common being feeling stressed, guilty, and worried. Feeling stressed was often related to the difficulty involved in having to manage competing tasks such as work, home-schooling/caring for children, and household chores. Additional sources of stress included increased workloads that led to less time for home-schooling and childcare, difficulty setting-up new routines for both parents and child, and the lack of unbroken time for work.

*I was feeling rather stressed about combining both work duties, household and home-schooling*. (M51; Part-time homeworking single-mother of one child).

Mothers with children with SEN had had to cope with the extra stresses stemmed from the delays in the implementation of educational and medical provision, unknowns around the transition back to school, and having their child’s needs met post-pandemic. The lockdown halted the progress in the delivery of services of vital importance for children’s wellbeing such as receipt of medication, assessment for developmental disorders, and associated funding for school-based support.

*He is on the waiting list of an autism assessment; I have been told it will be at least another 2 months before this goes ahead* via *Zoom. This affects the application for funding the school is making to try and ensure he gets the support he needs when he goes back in autumn*. (M25; Single- mother working from home full time, three children).

Feelings of guilt were related to various reasons including *not being good enough* (M06), not measuring up to other parents, not spending enough time/making an effort to home-school and support children’s learning. A few mothers reported feeling guilty for not being able to prioritise childcare over other competing demands. Other mothers were concerned of the effects of missing out on important educational experiences and the poor socialisation with peers, friends, and family members on children’s developmental outcomes. Guilt and worry around failing to support the child’s learning and academic achievement were also evident and expressed as *not being able to do get through work at the child’s pace* (M18), not putting a lot of effort helping with learning, not offering stimulation, and fear of children falling behind academically. A few mothers shared how they felt guilty and stressed for neglecting their children as they had to leave kids alone for long hours to be able to do their work.

*I feel guilty when I am taking care of my children that I am not working or keeping up with housework and when I work I feel guilty that my kids are watching too much TV*. (M42; Part-time homeworking partnered mother of two children).

*I felt I was rubbish at my job and at being a good mum as I could not do either well*. (M49; Full-time home working mother of one young child).

Many mothers were deeply worried over how changes in parenting and the family dynamic due to work pressures could influence child wellbeing and academic achievement. Work-related expectations and pressure to deliver triggered, for some, a chain of emotional reactions which were expressed as stress, family conflict, poor parenting behavior and associated feelings of guilt, worry and inadequacy.

*I have felt incompetent many times and stressed about keeping a routine for my son and do my job. I have also felt guilty for not meeting my son’s needs as I would expect so and also feeling like I am falling behind in terms of the quality of service I offer […]. All this pressure often manifests in arguments with my husband but also shouting at our son when we feel we have had enough which is something against our general parenting style. This of course creates more guilt and feelings of letting our son down but also worry about his development*. (M03; Full-time homeworking partnered mother of one child).

As well as guilt and worry over being a good parent, several reported similar feelings over being a good employee as a part-time homeworking partnered mother of two children (M28) reported.

*We do not have fiber so work has been very challenging with all 4 of us home so I’ve struggled with guilt of not being able to work as fast as others and anxious In case that is seen as not pulling my wait in uncertain job market.*

A few mothers experienced anger and resentment (i.e., *angry, upset, resentful, annoyed*). Often, these emotional states were a response to unequal distribution of home-schooling/ childcaring/ household chores, not being able to send kids to school like other parents, and increased workloads.

*I resent that because I earn less and work part time it is assumed I am willing and able to do all the childcare, home school and house care.* (M42; Full-time homeworking partnered mother of two children).

At times the negative emotions associated with H/CWW were evident even in a backdrop of positive maternal appraisals of the lockdown in terms of the opportunity to spend time with the children and bond with the family. For instance, the experience of a full-time homeworking single mother of three (M25) presented below offers an example of relishing the joys of working from home and spending time with children time while acknowledging the inherent difficulties in delivering home-schooling efficiently.

*I have been trying to deliver home-schooling in set periods so I can have set times I am at my desk but that has not always been feasible as I have been managing disagreements between my eldest two children. This has been frustrating at times, but it’s nice spending more time with the children, my daughter in particular. She would be asleep before I got home from work prior to lockdown and my eldest two* (*both boys*) *took my full attention at weekends so I did not get to spend a lot of time with her. Lockdown has meant we have been able to get to know each other and she is wonderful to play with, she’s so fun.*

Despite the difficulties, for a few mothers the experience evoked positive emotional states such as feeling grateful and motivated, although energy and motivation waned after a while for many. Of interest are the feelings of *pride* and agency that a few mothers experienced despite the difficulties. For a few mothers, notable were the feelings of happiness resulting from their passion for their work which was perceived as a source of financial independence and stimulation and provided a sense of purpose.

*I have been happy to be able to work as I enjoy the stimulation and financial independence*. (M42; Full-time homeworking partnered mother of two children).

*I have been very busy and it seems that I am working 24/7, but I love my job and I feel it gives me a feeling of doing something important*. (M08; Full-time homeworking partnered mother of one young child).

Single mothers shared similar emotions about H/CWW. Notwithstanding, the experience of not having anyone to turn to was unique to lone motherhood and may have exacerbated emotional distress in this population. For families where both parents were key workers, working shifts, meant that they too had to spend time with the children on their own. However, unlike single mothers they did not have to work while home-schooling/looking after children and be constantly available to meet children needs.

*The bad days were feelings of not coping, guilt for not being able to sit with my child and get through the work at his pace, overall guilt that nothing was being done properly because there was too much to do, feeling utterly exhausted and having no time to myself (not doing work or not tending to my child’s needs) and not being able to ask anyone for help*. (M18; Part-time homeworking single mother of one child).

### 4.3 Support

In partnered mothers having - or not having – support from their partner seemed to have influenced the experience of home-schooling and caring for children and associated emotions. We identified two models of paternal support. The first, involved the father taking up various levels of increased responsibility. Occasionally, this was the result of the father’s reduced workload or due to the increased demands for childcare which pressured some fathers to pick up some of the extra responsibilities that they did not use to be involved in before the pandemic.

*No real difference to be honest, as I usually work a lot from home My husband has taken up the majority of the home schooling to be honest, both as his work was significantly reduced, and because his workplace in the house is much closer to the children’s*. (M14; Part-time homeworking partnered mother of two children).

*It’s been hard. My husband has been doing more than usual (which was nothing) so it wasn’t impossible, but still hard*. (M33; Part-time homeworking partnered mother of two children).

In the second model, paternal support was provided in a context of collaboration and shared responsibility.

*Childcare and working from home is very hard. Having a supportive husband to share responsibilities with, helped a lot*. (M55; Full-time homeworking partnered mother of one child).

*It has been a stressful period. Good collaboration between me and husband was key. So arranging our schedule so that one of us has time to look after son*. (M12; Full-time homeworking partnered mother of one child).

However, a few reported a lower level of paternal involvement in childcare and home-schooling. Some of the reasons for a gendered approach to workload distribution included partner’s unavailability due to increased workload or because of protecting the time of the full-time partner which occasionally was the father.

*I’m having to insist on more equal leisure time as I’m struggling with planning home school, meal prep and have much less energy after work than he does*. (M21; Part-time homeworking partnered mother of one child).

*[…] my husband can ‘go to work’ in the spare room everyday and pretty much ignore us and be undisturbed whereas I am always multitasking*. (M42; Full-time homeworking partnered mother of two children).

*[…] (the lockdown) brought to light that the female is expected to take care of children more than men*. (M29; Part-time homeworking mother of four children).

*I’ve always had to juggle working full time and looking after the children (husband believes in gender specific roles!)*. (M30; Full-time homeworking partnered mother of two children).

It was not possible for the partners of single mothers to be involved physically during the lockdown. Moreover, conflict between ex-spouses regarding children’s upbringing and contact arrangements was another source of stress.

Flexible working arrangements including homeworking was another important source of support. Homeworking saved time from travelling to work. This, in turn, provided more time to spend with the family, to exercise and prepare fresh meals, to improve sleeping habits and, in some cases, to pick new hobbies.

*I do not miss rushing the children to get ready quickly to go to school so that I can be at work on time. I also do not miss being stuck in traffic and getting stressed out about being late*. (M30; Full-time homeworking partnered mother of two children).

*More sleep, less junk food, more exercise, more time with kids, more baking, less time at client meetings, more time with husband.* (M29; Full-time homeworking partnered mother of two children).

However, not everyone benefited by flexible working arrangements. A few mothers were worried over being less visible at work. For others it meant disrupted family routines, blurred boundaries between work and family life, and intensification of housework including preparing fresh meals and cleaning.

A few mothers reported that their workload and/or the work expectations decreased with employers being supportive, tolerant, and understanding. However, it is noteworthy that the workload increased dramatically for the majority of mothers in our sample. As a result, mothers had to deal with demands for extra work, on top of managing home-schooling/childcare. The increased pressures often were associated with negative implications for maternal and child wellbeing, maternal availability to support children’s learning, and work performance.

*Much more work needed - also much of this has been required in a rush. In the first three weeks I was working beyond midnight every single day (including weekends) and starting work around 6 am or earlier. This was required simply to keep up*. (M54; Full-time homeworking partnered mother of two children).

*Initially it was manageable but now after three months, the workload has increased a lot and it has affected negatively mostly working parents who have young children and cannot work in the same way as others who can sit down and concentrate uninterrupted during the day*. (M35; Full-time homeworking partnered mother of one child).

*[…] have struggled to combine this with my work sometimes, as there is an expectation that we are all constantly available while at work, giving little or no time to answer questions from the children, help, or even just male lunch*. (M14; Part-time homeworking partnered mother of two children).

The school was another important source of support and included provision of devices, individualized support for SEN, and access to lesson plans or worksheets posted online. On-line lessons were not always perceived as helpful and pleasant when combined with the expectation of sharing on-line parent-child joint activities which working mothers did not have the time to engage with. A few mothers voiced concerns over the adequacy of the support provided, the frequency of communication with teachers and schools, the excessive amount of homework, and the demands made on parent’s time to home-school. On a few occasions, the combination of limited school support and the daily stresses led to reduced expectations regarding children’s academic progress or giving up home-schooling.

*Doing everything was impossible. This is when I told the school that I would no longer be doing home schooling and if they decided to start zoom lessons to let me know and I would reassess. Until June, the work was simply worksheets posted onto a learning platform. No interaction from the school*. (M18; Full-time homeworking single-mother of one child).

*The daily Google classroom stream became quite oppressive - the ‘need’ to post delightful pictures of all the rich and exciting activities one was supposed to be doing was irritating. It was quickly dominated by the parents who were not working and those who were found it hard to engage (me included). That made it harder to handle*. (M54; Full-time homeworking partnered mother of one child).

There were mixed feelings about the use of social media such as Facebook as a source of supporting children’s home-schooling. Working mothers may have not been able to engage with the learning activities posted on social media due to time constraints. For a few mothers, the lack of engagement intensified the feelings of not supporting children’s learning good enough.

*I’ve found the constant stream of Facebook ideas of ‘how to home school your kids’ totally frustrating and at times I have felt very guilty that I am not spending the effort and energy that these other parents are home educating their kids and home made experiments etc etc*…. (M19; Full-time homeworking partnered mother of one child).

Finally, other sources of support included family members such as older children or relatives and neighbors. For a few mothers, the lockdown meant a heavier load of childcare as they could not rely anymore on the previously available support by others such as nannies, au pairs, and grandparents.

### 4.4 Coping strategies

Another common theme across partnered and single mothers was related to the strategies that they used to cope with the stresses of daily life as a result of H/CWW. These included adopting a positive and hands-on mentality that was manifested as acknowledgement and acceptance of the situation, and a *can-do* attitude.

*I thought it’s impossible to work and look after two kids at the same time but I can*. (M29; Full-time homeworking partnered mother of two children).

In the same vein, several mothers seized the opportunity for growth and transformation by reassessing priorities and life purpose, seeking meaning in the simplicity of life, practicing gratitude for good health, and through patience. Others rose above the adversity through spiritual practices reflected in connecting with nature and living in the moment.

*It’s been a simpler life, more focus on the “mum” role which has been lovely*. (M11; part-time homeworking single mother of three children).

*My thoughts and feelings were that we should be grateful that we are healthy and well and we should be patient until all this comes to an end*. (M40; Full-time homeworking partnered mother of three children).

*This was stressful and hard for body and mind but at the same time I was feeling so blessed for my healthy family. I tried to live “the here and now” and concentrate on play with my son. This gave him confidence and made me feel even better*. (M15; Part-time homeworking partnered mother of one child).

Flexibility was key in overcoming emotional strain. It involved focusing on what works for family wellbeing in the context of the new challenges, on changing family practices and routines no longer serving the family and creating new ones that fostered family resilience. These adaptations were expressed as reviewing expectations in terms of housekeeping, time spent with kids, work performance, amount of schoolwork completion, and adhering to a rigid schedule such as bedtime and screen time.

*Started quite stressful trying to fit everything in, we have gradually let certain things slip and that’s removed some of the stress. Does it matter if the kids aren’t studying by 9 am? Does it matte(r) if the floors have not been washed for a week, does it matter if the kids miss showers or baths on some days. Some trade-offs I guess. I did start to intensify become adverse to lockdown motivators, I did not have free time to learn something new or make my house sparkle, but now I realise it was people just getting by!* (M16; Full-time homeworking partnered mother of two children).

Others focused on self-organization and management including establishing a family routine, and planning activities to keep the children busy while working. In the context of two-parent families, negotiating clear boundaries, collaboration, and allocation of responsibilities in a spirit of fairness, such as setting a schedule of childcare duties to share between partners, allowed to work more efficiently. Connectedness with the family and community of parents with similar experiences was another important strategy that kept several mothers afloat. Amidst the difficulties with home-schooling/childcare a few mothers saw the opportunity that school closures and remote working offered to make up for the lost time with the family and learn their children better.

*I have zero time to myself so getting stuff done has been difficult but I love that I get to spend so much time with the children - I’ll probably never have this opportunity again so am trying to enjoy it whilst it lasts*. (M4; Full-time not working from home partnered mother of two children).

### 4.5 Word frequency analysis for emotional states

**Figure 1:**
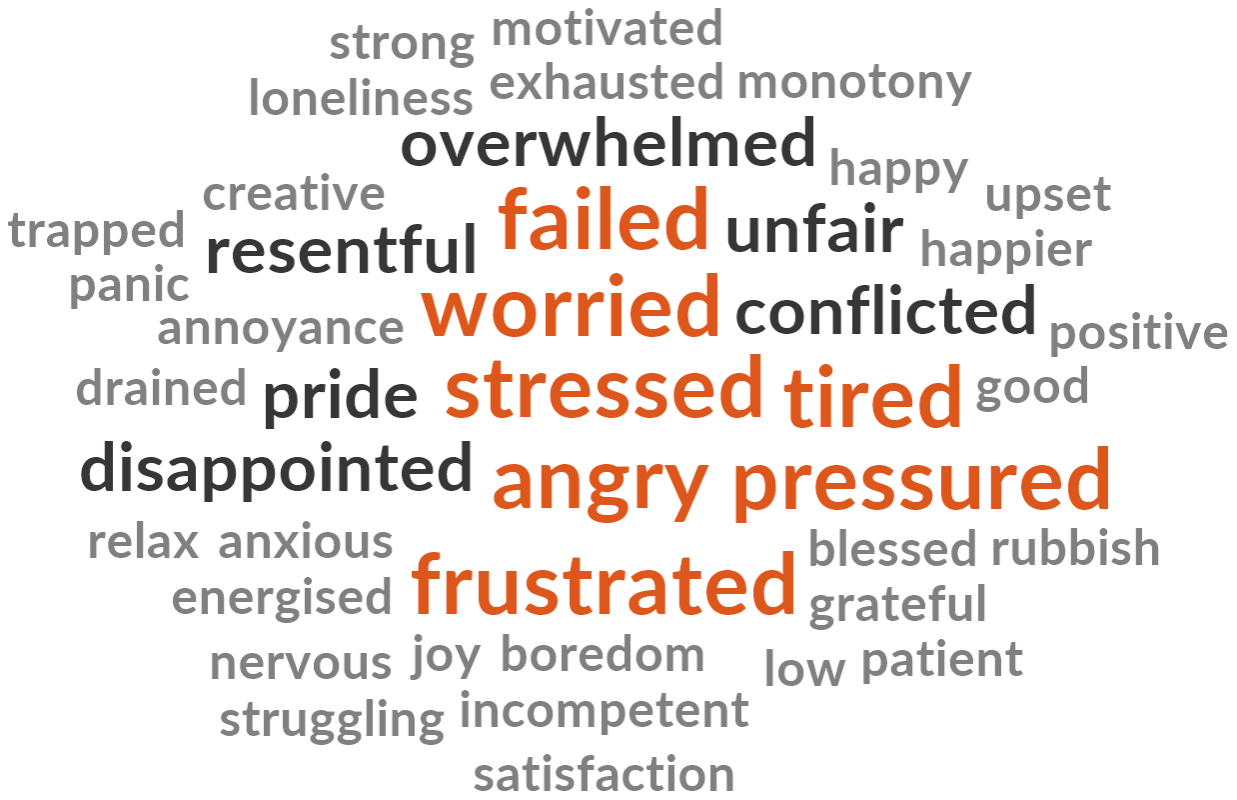
Word cloud of maternal emotional states while home-schooling and/or caring for children during the COVID-19 lockdown in the United Kingdom.

The first author screened the maternal excerpts used to inform the thematic analysis for words that reflected emotional states (e.g., *sad*/*sadness*). First, the words identified were subject to a word frequency analysis using NVivo 12. This process yielded a list of 45 words (word frequency range: 9–1). If a word appeared more than once in the excerpts of the same individual it still counted only as one toward the total word count. Words with the same meaning (e.g., guilt/guilty) appeared as one word (e.g., guilty). We could not subject into the analysis sentences. Second, we cleaned the list of words by adjusting for spelling mistakes (e.g., ungry for angry). Additionally, we used the on-line Cambridge dictionary^3^ to identify synonyms for expressions and sentences that we could not use in the word frequency analysis. These include the expression *felt hard-done by* which refers to *feeling treated unfairly*. Accordingly, we replaced it with the word *unfair*. We replaced the expression *not good enough* with the word *inadequate* because it is synonym to *not adequate.* To replace the expression *felt not coping* we looked for the synonym of the word *cope* which is *manage*. Then we looked for the antonym of manage which is *fail* and used it to replace the expression. We ended up with a list of 43 words (Supplementary file 2). The word frequency analysis indicated that the most common emotional states reported were feeling *stressed* (*n* = 9), *guilty* (*n* = 8) and *worried* (*n* = 4) followed by *frustrated*, *pressured, tired*, *angry* and *failed* (*n* = 3), *conflicted*, *disappointed, pride, resentful, overwhelmed* and feelings of being tread unfairly (*n* = 2). The words were subjected to a word cloud analysis presented in Figure 1. The analysis confirmed the findings from the thematic analysis in terms of the most common emotional states shared by the mothers in our sample: stress, guilt, and worry.

## 5 Discussion

COVID-19 related mitigation measures like lockdowns placed significant burdens on parents, who found themselves simultaneously caring for and homeschooling their children while managing work responsibilities. Mothers were said to have experienced important mental distress because of shouldering most of the extra caregiving and home-schooling. The study aimed to explore the homeworking and childcare experiences of working mothers during the United Kingdom COVID-19 lockdown, and their emotional and practical responses to these experiences.

One of the main findings was that having to meet the responsibilities of four competing roles at the same time, that of the professional, mother, housekeeper, and teacher, was a strenuous and anxiety-inducing experience. The experience was taxing even for the mothers who shared positive lockdown experiences such as bonding with family and flexible working arrangements. Mothers have been historically fulfilling multiple roles simultaneously; however, the confluence of responsibilities deriving from the task of home-schooling and tending to their children while shouldering professional obligations inflicted considerable strain upon them. There is substantial heterogeneity in the effects of the lockdown on the well-being of both parents and children according to background variables (Brooks et al., 2020; Crisci et al., 2021). Accordingly, we found that the quality of the experience of H/CWW varied substantially based on maternal personal circumstances and child characteristics. For instance, a mother’s balancing act of fulfilling multiple roles could be severely compromised by maternal lack of confidence with home-schooling and the child’s receptiveness to home learning. Our findings warrant the investigation of the interaction between maternal personal characteristics and the maternal microsystem of influences in understanding better maternal emotional wellbeing during and after the pandemic. The findings indicated that reducing expectations regarding children’s academic learning or giving up home-schooling was one of the ways to deal with the pressure imposed by the laborious task of H/CWW. However, varying degrees and quality of access to home learning during the lockdown could have deepened societal inequalities (Andrew et al., 2020c). In the event of a future public health crisis necessitating the implementation of quarantine measures, the opportunity to support children’s home-schooling in parents’ own time could be a more sustainable solution for working families and may increase access to home learning.

Several mothers found coping with the teacher role extremely tough due to their low confidence in their skills to support their children’s learning and perceived incompatibility with the mothering role. Contrary to our findings, a study of middle-class partnered working mothers living in Hungary and Romania during the lockdown suggests that maternal experiences reflected important pride and agency in supporting children’s learning (Somogyi et al., 2022). It is not clear what causes the difference. One plausible explanation is the different level of access to teacher support during the lockdown across countries. Parents in the United Kingdom were reported to be amongst the least likely to agree that children had contact with a teacher (Thorell et al., 2021). On the contrary, in Hungary online schooling was implemented from day one of the lockdown (Somogyi et al., 2022). Working mothers in the United Kingdom had to become teachers overnight while having to juggle work and household responsibilities. Home learning responsibilities might have been intensified for parents in the United Kingdom, if support with remote schooling was limited. Another possible explanation is school and societal expectations about parental involvement in children’s education. Parents choose to be involved in their children’s schooling based on school expectations for involvement (Hoover-Dempsey and Sandler, 1995). Moreover, societal expectations about the level of maternal involvement in children’s learning may vary across countires. Future investigation of working mothers’ perception of the teacher role during the lockdown should consider the school support received and school and societal expectations of parental involvement in children’s education.

As well as revealing the quality of maternal experiences of H/CWW, our study unearthed the associated emotional load. Stress because of the difficulty that comes with meeting the demands of multiple and conflicting roles, that of the worker, teacher, carer, and housekeeper, was one of the most reported emotions. The role strain theory proposes that the higher the pressure to fulfil multiple roles or to meet more demands within a role the higher the likelihood to experience role strain (Goode, 1960). The findings suggest that the mothers in our sample experienced a high level of stress because of role strain. Stress over the impact of synchronous home-schooling and childcare on work performance was common too. Additionally, mothers felt guilty for failing to attend to their children’s needs because of having to prioritize work over childcare and/or home-schooling. Worry often over the impact of maternal unavailability, low involvement in children’s learning, and poor parenting behavior, was another common emotional state. Significant anxiety and worry were experienced by mothers with children with SEN because of the delays in provision implementation and the uncertainty about what the future holds in the post-COVID-19 era. Additionally, stress over not having no one to turn to was reported by lone mothers. Our findings confirm earlier contentions that the challenge of balancing paid work and childcare have had a negative impact on maternal mental wellbeing (Andrew et al., 2020c). Persistent and consistent feelings of stress, anxiety and worry are indicators of poor mental health (Sánchez-Rodríguez et al., 2019). Moreover, guilt is common in mothers who report emotional and physical burnout (Hubert and Aujoulat, 2018). Feelings of inadequacy, worries over not fulfilling one’s parental duties, and the internal conflict about prioritizing work over the parenting role and vice versa are relevant to the societal pressures to be the perfect parent while managing the household, keeping a paid job, and pursuing career aspirations (Meeussen and Van Laar, 2018). Pressures from unrealistic expectations can put mothers at risk of mental health difficulties (Henderson et al., 2016; Lamar et al., 2019), and jeopardize their career ambitions (Meeussen and Van Laar, 2018). Because H/CWW was a situation that specifically affected working mothers, the associated emotional distress could be a unique predictor of mental health during the pandemic among working mothers.

Research on family stress and its theoretical underpinnings emphasize the negative impact of the lack of formal or informal support on parental wellbeing which adversely influences parenting and eventually leads to poor child outcomes (Armstrong et al., 2005). The analysis showed that maternal experiences and associated emotions were nested within an enabling or disabling system of informal and formal support which influenced their mental health and wellbeing. Three major sources of support were identified: paternal, work and school support. While some fathers increased their involvement, in some households a gendered approach to domestic work was applied. Our findings agree with research showing that despite paternal engagement in child and family care rising significantly during the pandemic many mothers spent significantly more hours on home-schooling and caring for children even when they were working (Craig and Churchill, 2020; Andrew et al., 2020b). A few mothers reported difficulties managing a heavier workload and feelings of resentment as a result of unequal distribution of domestic responsibilities. Our findings align with earlier research which showed that gender differences in unpaid care work are associated with poor psychological wellbeing (Ausín et al., 2021; Xue and McMunn, 2021). We did not find any evidence of positive paternal involvement in lone parent households.

As well as the importance of paternal time spent in supporting children’s learning and care, our data emphasize the nature of paternal engagement that can make a meaningful contribution to eliminating the gender gap in time invested in child upbringing. Our data revealed that uninterrupted time to work was reported as one of the most challenging and emotionally taxing aspects of the lockdown. Additionally, lack of uninterrupted work time because of unequal distribution of domestic work co-existed with feelings of resentment and frustration. During the lockdown mothers not only did they increase overall the hours spent looking after the children but ended up doing so during work hours a lot more often than fathers did (Andrew et al., 2020a). Working while taking care of other tasks can negatively influence maternal mental health as it can increase anxiety and worry over one’s productivity (Andrew et al., 2020a). Differences in productivity might lead to increases in gender inequality in the next few years, particularly in high-skilled jobs where promotion tracks are important (Ucar et al., 2022). Unlike fathers, mothers are quicker to adjust their work patterns to meet the needs of the family. Fathers are more likely than mothers to follow expectations of the ‘ideal worker’ unobstructed by domestic responsibilities; on the contrary, mothers are likely to conform to expectations as homemaker and caregiver even if they are employed (Zanhour and Sumpter, 2022). The home-schooling experience has provided a unique opportunity to employers to reconcile with the fact that having a family comes with responsibilities. The assumption that having a career precludes family responsibilities is not sustainable in a time of rapid societal change (Alon et al., 2020).

Flexible working arrangement including remote working and less pressure to be available during home-schooling hours were often reported together with positive experiences. Remote working came with several important benefits for family relationships and child education including the opportunity to spend more time with the family, and higher parental involvement in children’s schooling. For some mothers flexible working meant better mental and physical wellbeing as a result of better sleep quality and cutting down stressful commute to work. On the other hand, for some mothers remote working was associated with more blurred work life boundaries, and less unbroken time to work. As hybrid working has become the new standard post-pandemic, special care by governments and employers should be taken to protect the career prospects and work-life balance of working mothers. Additionally, the findings propose the adoption of a less rigid conceptualization of flexible working to allow the consideration of a greater pool of work models. These will be determined by individual need and could include flexibility around both the time and place of work. For many mothers around the world school closures were *a blessing in disguise* (Abrefa Busia et al., 2022). The combination of flexible working arrangements and the lockdown gave them the opportunity to regain the lost time with their children and evade the stresses that come with daily routines at home and work. However, for most mothers in our sample, this was hindered by the increased workloads which might have put some working mothers at higher risk of poor mental health and even counteracted the benefits of remote working.

Regarding school support we identified various types of support that were helpful including on-line lessons and home-school communications. These were often associated with positive home-schooling experiences. A few mothers reported dissatisfaction with school support which included lack or insufficient on-line teaching, poor communication between school and family, and a large volume of taught material. For some mothers these troubles were compounded by poor infrastructure (e.g., inadequate number of devices and/or poor internet connection), a key ingredient for the success of remote learning (Education Endowment Foundation, 2020). Additionally, factors such as the child’s SEN, age and temperament, and maternal characteristics such as lone parenthood, and low maternal confidence in supporting children with home-schooling impacted on the quality of the homeschooling experience. The research on school closures on student outcomes warned about how varying degrees and quality of access to home learning would deepen education inequalities and called for research in to the factors implicated (Grewenig et al., 2021). Our findings revealed some of the school factors that could be considered in future investigations on child access to remote learning. Maternal concerns over child wellbeing were overwhelming so several mothers did not homeschool to relieve some of the pressure. This finding emphasizes the importance of child social and emotional wellbeing for maternal wellbeing and stress-free and thriving family life. Together with physical health, mental health lay the foundation from which children and young people can start exploring the learning opportunities available to them (Weisbrot and Ryst, 2020). Post pandemic the aims of education may have to be re-examined, and school provision could consider integrating academic as well as social and emotional learning goals more systematically in the curriculum.

As well as systems of support, the capacity for positive adaptation to stressful situations has protective qualities for maternal wellbeing (Jones et al., 2022). The findings help to understand better what resilience looked like among working mothers in the context of H/CWW during COVID-19 as well as more generally in the face of adversity. Specifically, positive emotional experiences often co-existed with a range of coping strategies that reflected aspects of the three key resilience processes outlined in Walsh’s (2003) work of family resilience: family belief systems, organizational patterns and communication/problem solving. The findings showed that several mothers adhered to family belief systems that help make meaning of crisis and focus on problem resolution, healing and growth. Examples include adopting a positive perspective to adversity, acceptance, a desire for transformation and growth from adversity, and the adoption of ritualistic routines to help reduce the stress. Family organization systems that promote family resilience were reflected in efforts to adopt flexible family structures including openness to embrace change of well-established routines, and increased family connectedness. Additionally, in the context of partnered mothers, new family organization systems emerged in the form of new models of partnership that allowed cooperation between partners in home-schooling and childcare. Finally, reframing of the problem by focusing on the opportunities offered instead, was a problem-solving strategy used by a few mothers. These resilience characteristics can be targeted and enhanced by resilience interventions for working mothers in the event of a crisis or at times of adversity. Nevertheless, resilience comes with great heterogeneity in people’s responses to environmental diversity (Rutter, 2012); not all mothers shared coping strategies and there were cases of mothers who shared their strategies in a context of intense emotional frustrations. These findings suggest that a research priority in understanding mental wellbeing of working mothers during the lockdown and during adversity should adopt a multisystemic approach that considers several factors of influence on the frequency and type of coping strategies adopted.

### 5.1 Limitations

The study had some methodological limitations. Because the sample comprise primarily white mothers with a higher education caution is required when generalizing to the wider population of working mothers. Nonetheless, that negative consequences of H/CWW were found to be more acute amongst women with higher education (Fodor et al., 2021). Therefore, our findings are relevant to the population that is most likely to have been affected. Because the survey might have been accessed by mothers who were feeling particularly overwhelmed and distressed with the extra responsibilities, maternal impressions and emotions could be exaggerated. For instance, professional and highly educated mothers might have been more likely to have lost external childcare support, to have high flying career-oriented partners and therefore contribute more to childcare or apply intensive mothering which is labor-intensive (Fodor et al., 2021). While we should be cautious extrapolating to the wider population of working mothers in the United Kingdom, understanding the experiences of those who struggled the most can inform the design of targeted interventions. Our findings were not triangulated with quantitative or multiple methods of data collection but having two coders contributed to enhancing the validity of the analysis and the findings. The study does not include fathers’ perspectives. As participants were asked to respond to open ended questions, we were not able to ask more in-depth questions that could shed light into the underlying processes affecting mothers’ experiences and wellbeing during the COVID-19 lockdown. However, the survey method of open-ended questions allowed to sample a wider range of experiences in a short period of time.

## 6 Conclusion and implications

The study findings showed that the H/CWW experience during the first United Kingdom COVID-19 lockdown was practically and emotionally challenging for both partnered and single working mothers. Working mothers’ emotional experiences varied depending on child and mother characteristics, the range of practical supports available to them and the strategies they used to cope with the challenges of H/CWW. The study suggests that improved access to support systems provided by work and school could support maternal wellbeing in future lockdowns or at times of adversity. Workplace related support include more flexible working arrangements, manageable workloads, and the cultivation of a workplace culture that promotes employee health and wellbeing. School supports includes timely access to online learning and relevant infrastructure, closer school-family communication, manageable taught material that can be delivered by parents, provision for families with children with special educational needs and single-parent households. As well as access to school and work support, increased paternal involvement in childcare and homeschooling could positively contribute to maternal emotional wellbeing during a lockdown. These findings underscore the need to address the gender norms that assign uneven responsibility for domestic and care work on women and to promote family-friendly working policies that facilitate paternal involvement in family life. Several of the strategies used by the study participants reflected skills that are targeted by mindfulness training which focuses on attending to the present in a non-judgmental or accepting way (Baer et al., 2004). Mindfulness-based interventions have been found to be useful in improving mental health particularly symptoms of depression (Duarte et al., 2019). In the event of future lockdowns easy, and timely access to low intensity mindfulness based psychological therapies could support maternal wellbeing.

## Data availability statement

The raw data supporting the conclusions of this article will be made available by the authors, without undue reservation.

## Ethics statement

The studies involving humans were approved by the Ethics Committee at the University of Turku, Finland, Humanities and Social Sciences Division. The studies were conducted in accordance with the local legislation and institutional requirements. The participants provided their written informed consent to participate in this study.

## Author contributions

AK: conception and study design, data collection and analysis, write-up of first draft and submitted version, revisions. P-ZT: study design, questionnaire design, data analysis, and write-up of sections of first draft. All authors contributed to manuscript revision, read, and approved the submitted version.

## Footnotes

^1^https://webropol.com/

^2^https://lumivero.com/products/nvivo/

^3^https://dictionary.cambridge.org/
