# Supplementary material for: Home-schooling and caring for children during the COVID-19 lockdown in the UK: emotional states, systems of support and coping strategies in working mothers
Source: Front Sociol. 2024 Mar 21;9:1168465. doi: 10.3389/fsoc.2024.1168465 (PMC10991830; doi:10.3389/fsoc.2024.1168465)
Supplement: Supplementary file 1 [file Data_Sheet_1.PDF]

Pandemic Mood 2020: the views of working and studying mothers about coping with the challenges of the state of emergency

Dear working / studying mother,

We invite you to answer our survey related to the main thoughts and feelings you have experienced during this pandemic. The aim of the survey is to clarify whether the well-being of working/studying mums has changed during the state of emergency caused by Covid-19. We are interested in how the state of emergency has changed your daily life as well as how those changes have affected your mood. For instance, how have you experienced combining work/studies, childcare/home schooling and house chores, and what kind of thoughts and feelings have those aspects brought to you.

The survey consists of background information and six open-ended questions that target the following themes: general mood, work/studies, social life, daily life, and combining work and home during the state of emergency as well as thoughts regarding slowly opening up the society again.

All the information you give on the survey form will be handled confidentially and with anonymity. We will not ask your name or other very personal information that you could be recognized from, and no individual respondents can be identified from the data at any point of the research. The study follows the ethical guidelines of the Finnish National Board on Research Integrity (TENK), and the privacy statement of the research can be accessed from the link below.

Privacy statement

You can contact the responsible researchers for more information about the study:
xxxxx

Thank you for your valuable contribution!

1. I have read the privacy notice and I agree to participate in the survey. *

Yes

○

2. Your age *

____________________ 2 characters left

3. Your working situation *

- ◯ Full-time work
- ◯ Part-time work
- ◯ Studying
- ◯ Other, describe (if you work in a university please say so) [ ]

4. Highest educational level completed *

- ◯ Complete GCSE/CSE/O-levels or equivalent (at school till age 16)
- ◯ Completed post-16 vocational course
- ◯ A-levels or equivalent (at school up to age 18)
- ◯ Undergraduate degree or professional qualification
- ◯ Postgraduate degree

5. Ethnic background *

- ◯ White - British, Irish, Other
- ◯ Black/Black British - African, Carribean, Other
- ◯ Asian/Asian British - Indian, Pakistani, Bangladeshi, Other Asian
- ◯ Chinese/Chinese British
- ◯ Mixed race - White and Black/Black British
- ◯ Mixed race other
- ◯ Middle Eastern/Middle Eastern British - Arab, Turkish, Other
- ◯ Other ethnic group
- ◯ Prefer not to say

6. Number of children *

______________________ 10 characters left

7. Age of each of your children *

______________________ 50 characters left

8. Please describe your current family structure (e.g. two-parent family, cohabiting partnership-in case your partner is not the father of your kid/s, single motherhood, other).
If not at the same household, is the father of your child/children involved in their care and remote schooling (if applicable)? *

500 characters left

9. Your current workplace during the state of emergency *

- ◯ Working / studying from home
- ◯ Working / studying outside the home

10. The current workplace of your partner during the state of emergency (if applicable)

- ◯ Working / studying from home
- ◯ Working / studying elsewhere than home

11. During the state of emergency, your children have been *

- ◯ At home (home care, remote schooling)
- ◯ Going to school /preschool /daycare
- ◯ Other, describe below (e.g., one child homeschooled, other child in daycare, etc.) [ ]

12. Do you or members of your immediate family (partner, child) belong to any of the covid-19 risk groups? *

- ◯ Yes
- ◯ No

13. Country of residence during the state of emergency *

During the state of emergency caused by the covid-19 pandemic...

14. How has your mood been in general?
What have your main thoughts and feelings been?

2500 characters left

During the state of emergency caused by the covid-19 pandemic...

15. How has working / studying from home (or working outside the home during the pandemic) affected your mood and feelings? (e.g., the mode of work, changes in the amount of workload, and/or other)

2500 characters left

During the state of emergency caused by the covid-19 pandemic...

16. How has your social life changed? How have the changes in your social life affected your mood and feelings? (e.g., meeting friends, group activities, social events, and/or other)

2500 characters left

During the state of emergency caused by the covid-19 pandemic...

17. How has your everyday life changed? How have those changes affected your mood and feelings? (e.g., daily routines such as meal times, sleeping quality and sleeping schedule, physical activities/ exercising, leisure time and/or other)

2500 characters left

During the state of emergency caused by the covid-19 pandemic...

18. How have you experienced the combination of (remote) work, managing kids' remote schooling (if applicable), and child care? What factors have made it easier and/or harder to handle? What thoughts and feelings has this situation brought to you?

2500 characters left

During the state of emergency caused by the covid-19 pandemic...

19. Now that the society is starting to slowly open up again, what kind of thoughts and feelings has it brought to you? How has it affected your mood?

2500 characters left

20. Feel free to add anything that was not mentioned and you feel important to share / clarify.

2500 characters left

21. Would you like to receive news of the project as it progresses (research results, information about publications etc.)? If so, please leave your email address in the text field below.
Note! The email address will not be combined with your answers and will not be used for any other purpose than for sending information regarding the progess of the Pandemic Mood study.

____ 100 characters left
