# Supplementary material for: Home-schooling and caring for children during the COVID-19 lockdown in the UK: emotional states, systems of support and coping strategies in working mothers
Source: Front Sociol. 2024 Mar 21;9:1168465. doi: 10.3389/fsoc.2024.1168465 (PMC10991830; doi:10.3389/fsoc.2024.1168465)
Supplement: Supplementary file 2 [file Table_1.DOCX]

|  | Word | Count |
| --- | --- | --- |
| 1 | stressed | 9 |
| 2 | guilty | 8 |
| 3 | worried | 4 |
| 4 | frustrated | 3 |
| 5 | pressured | 3 |
| 6 | tired | 3 |
| 7 | angry | 3 |
| 8 | failed | 3 |
| 9 | conflicted | 2 |
| 10 | disappointed | 2 |
| 11 | pride | 2 |
| 12 | resentful | 2 |
| 13 | overwhelmed | 2 |
| 14 | unfair | 2 |
| 15 | annoyance | 1 |
| 16 | anxious | 1 |
| 17 | blessed | 1 |
| 18 | boredom | 1 |
| 19 | creative | 1 |
| 20 | drained | 1 |
| 21 | energised | 1 |
| 22 | exhausted | 1 |
| 23 | good | 1 |
| 24 | grateful | 1 |
| 25 | happier | 1 |
| 26 | happy | 1 |
| 27 | incompetent | 1 |
| 28 | joy | 1 |
| 29 | loneliness | 1 |
| 30 | low | 1 |
| 31 | monotony | 1 |
| 32 | motivated | 1 |
| 33 | nervous | 1 |
| 34 | panic | 1 |
| 35 | patient | 1 |
| 36 | positive | 1 |
| 37 | relax | 1 |
| 38 | rubbish | 1 |
| 39 | satisfaction | 1 |
| 40 | strong | 1 |
| 41 | struggling | 1 |
| 42 | trapped | 1 |
| 43 | upset | 1 |
